# Potential for a cerebellar role in moderate-late preterm associated behavioural disorders

**DOI:** 10.3389/fped.2024.1336137

**Published:** 2024-01-26

**Authors:** Carlton L. Pavy, Julia C. Shaw, Roisin A. Moloney, Hannah K. Palliser, Jonathon J. Hirst

**Affiliations:** ^1^ School of Biomedical Sciences and Pharmacy, University of Newcastle, Newcastle, NSW, Australia; ^2^ Hunter Medical Research Institute, Mothers and Babies Research Centre, Newcastle, NSW, Australia

**Keywords:** cerebellum, preterm birth, myelination, neurosteroids, hypoxia, neurodevelopment

## Abstract

Preterm birth is known to cause impaired cerebellar development, and this is associated with the development of neurobehavioral disorders. This review aims to identify the mechanisms through which preterm birth impairs cerebellar development and consequently, increases the risk of developing neurobehavioral disorders. The severity of these disorders is directly related to the degree of prematurity, but it is also evident that even late preterm births are at significantly increased risk of developing serious neurobehavioral disorders. Preterm birth is associated with hypoxic events and increased glutamatergic tone within the neonatal brain which contribute to excitotoxic damage. The cerebellum is a dense glutamatergic region which undergoes relatively late neurodevelopment up to and beyond birth. Evidence indicates that the cerebellum forms reciprocal connections to regions important in behaviour regulation such as the limbic system and frontal cortex. Studies using fMRI (functional magnetic resonance Imaging), BOLD (blood oxygen level dependent) response and morphology studies in humans show the cerebellum is often involved in disorders such as attention deficit hyperactivity disorder (ADHD) and anxiety. The vulnerability of the cerebellum to preterm birth insult and its connections to behaviour associated brain regions implicates it in the development of neurobehavioral disorders. Protection against preterm associated insults on the cerebellum may provide a novel avenue through which ADHD and anxiety can be reduced in children born preterm.

## 1 Introduction

Globally, 10% of births are preterm and ∼90% of these are moderate-late preterm births (32–36 weeks completed gestation) (1, 2). Factors leading to the onset of preterm birth are still poorly understood, with many aetiologies and currently no treatments effective for prevention. Moderate-late preterm birth is associated with decreased executive functioning in later life and an increased risk of developing neurobehavioral disorders compared to term births (3–7). Executive function underlies complex cognitive processes and deficits can greatly affect an individual's ability to learn, socialise, develop relationships and regulate emotions (8, 9). Extensive evidence indicates that ADHD and anxiety begin to develop in school aged children with ∼5%–10% of school aged boys being diagnosed with ADHD and ∼10% of all children being diagnosed with an anxiety disorder (10, 11). ADHD and anxiety both have approved pharmacological treatments although these treatments have a suite of contraindications and adverse effects. Compliance is another major limitation of current treatments which require long term application, and often, ongoing specialist paediatric consultations. In addition, evidence for their ability to alter the long-term trajectory of these disorders is limited (12–14). Current therapies should be regarded as symptomatic treatment as they do not address or reverse the neurodevelopmental changes that occur in late pregnancy. Currently there are no treatments which address the insults that occur in the early neonatal period following preterm birth.

It is well established that the cerebellum is critical in motor-related predictive processes although more recent literature has found the cerebellum is a hub of cognitive prediction which can regulate the speed, capacity, consistency and appropriateness of mental or cognitive processes (15, 16). Both ADHD and anxiety have been associated with cerebellar dysfunction and this review aims to outline the mechanisms through which preterm birth can impair the development of the cerebellum and result in the phenotypic behaviours of these pathologies (Table 1). This review includes information which was found using Pubmed and only studies which were written in English have been included.

**Table 1:** Key studies from this review with brief outline of methodology, results and relevance.

Reference	Sample species and methodology	Results	Relevance
Walsh et al., (48)	Human, MRI, Preterm vs. Term	Preterm birth caused significantly decreased cerebellar myelination.	Strong evidence of preterm birth impairing cerebellar myelination.
Zonouzi et al., (53)	Mouse, hypoxia exposure treated with tiagabine, assessed by IHC	Hypoxia caused reduced oligodendrocyte maturity. Tiagabine administration restored oligodendrocyte maturation.	Demonstration of hypoxia's ability to impair oligodendrocyte maturation. Good evidence of GABAergic therapies protecting against hypoxic/excitotoxic damage.
Ivanov et al., (65)	Humans, MRI, ADHD vs. control	ADHD resulted in decreased cerebellar surface and underlying tissue volumes.	This study shows clearly that ADHD and cerebellar abnormalities are linked.
Potjik 2012	Human cohort study (>1,000 children), comparing moderate-late preterm with term born children.	Moderate-late preterm born children had sex dependent increases in internalising behaviour score (females) and externalising behaviour scores (males)	This study demonstrates the impact moderate-late preterm birth has on childhood behaviour phenotype.
Vacher et al., (96)	Mice, genetic knockdown of allopregnanolone placental supply, treated with allopregnanolone replacement. Assessed by IHC and behaviour tests.	Reduced allopregnanolone resulted in decreased cerebellar myelination and an autistic-like phenotype. This was prevented in mice treated with allopregnanolone.	Strong evidence that allopregnanolone is involved in cerebellar development and that impairments can greatly impact behaviour. This study further demonstrates the potential benefits of interventions which utilise neurosteroid pathways.

## 2 Hypoxia risk in the preterm neonate

**Figure 1:**
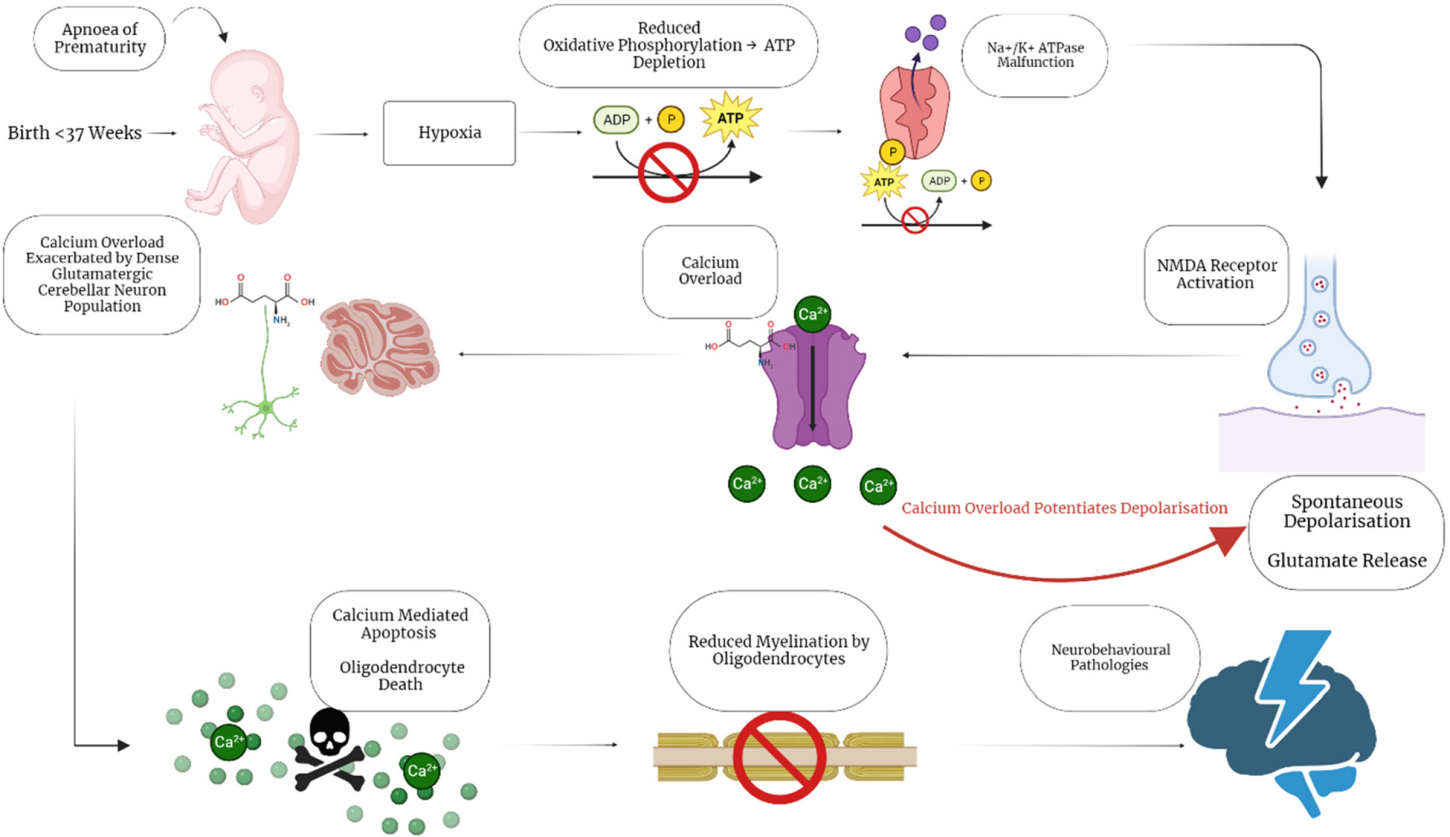
Hypoxic/excitotoxic cascade impairs myelination following apnoea of prematurity. Preterm birth is associated with apnoea of Prematurity, a condition which causes neonatal hypoxia. Neonatal hypoxia sets off a cascade of events that illicit calcium overload through spontaneous depolarisation of glutamate receptors. The calcium overload is exacerbated by the large population of excitatory, glutamatergic granule cells in the cerebellum, resulting in calcium mediated apoptosis. Oligodendrocytes are particularly sensitive to these insults, resulting in reduced mature oligodendrocytes and myelination disruption. The impaired myelination then further contributes to the development of neurobehavioral pathologies by impairing neuronal connectivity and signalling. Created with BioRender.com.

Preterm neonates are at increased risk of neonatal brain injury due to their exposure to hypoxic/ischaemic events. Hypoxic events are known to occur very commonly in preterm neonates due to their cerebral vascular fragility, irregular blood pressure, immature lungs and exposure to resuscitation (17–19). Additionally, preterm neonates also face the risk of experiencing apnoea of prematurity. Apnoea of prematurity is defined as respiratory pauses >20 s or pauses <20 s that are associated with bradycardia (<100 beats/minute), central cyanosis, and/or oxygen saturation <85% in neonates born at <37 weeks gestation. Reduced respiration in neonates experiencing apnoea results in a heightened risk for hypoxic damage as well as driving excitotoxic damage in the brains of these neonates. Hypoxia drives excitotoxic events as reduced oxygen causes oxidative phosphorylation to become impaired, preventing neurons from producing ATP. ATP dependent ion pumps then begin to malfunction, resulting in spontaneous release of glutamate by synaptic vesicles as well as impairing glutamate reuptake (20). The combination of apnoea of prematurity with the respiratory and cerebrovascular immaturity present in preterm neonates result in a high risk of preterm neonates experiencing excitotoxicity, which is known to result in brain injury (Figure 1).

## 3 Preterm birth particularly increases the risk of damage to the cerebellum

Preterm birth is the leading cause of perinatal brain injury and impairment of neurodevelopment. In humans most cortical neurogenesis is completed by mid-gestation but this does not represent neurodevelopment being completed (21–23). Neuronal migration, synapse formation, synapse pruning, programmed apoptosis and myelination continue far beyond mid-gestation even into neonatal life (23, 24). Brain regions including the cerebellum are still undergoing extensive neurodevelopment during late gestation and into postnatal life. The volume of the cerebellum increases by ∼3.5 fold from 28 to 40 weeks gestation in humans. Rapid cerebellar growth during the period corresponding to preterm birth suggests a high vulnerability of the cerebellum to preterm birth insults (25). Factors provided by the *in-utero* environment are crucial in maintaining optimal development of multiple processes and premature loss of these conditions can drastically impair this process, resulting in damage to the cerebellum.

The cerebellum is a highly neuron dense brain region which comprises ∼10% of the brains volume whilst contributing ∼ of the brain's neuron population. Most of this neuron population is made up of the glutamatergic, excitatory, cerebellar granule cell (26, 27). These cerebellar granule cells constitute half the population of neurons in the entire human brain. Within the cerebellum, granule cells outnumber the GABAergic, Purkinje cell 2000:1, resulting in a highly dense excitatory environment (28). As discussed above, oligodendrocyte progenitor cells have been found to be especially vulnerable to excitotoxic damage (29, 30). The cerebellum also shows a ratio of ∼0.23 glia to neurons vs. ∼11.35 in the rest of the brain (31, 32). Hypoxia-ischemia is common following preterm birth and this leads to glutamate excitotoxicity. Astrocytes are responsible for the majority of uptake of glutamate in the CNS (80%–90%) and the low ratio of glia to glutamatergic neurons in the cerebellum suggests that glutamate release could more readily reach excitotoxic levels which would be especially impairing given the relatively small population of oligodendrocytes within the cerebellum (33, 34). Purkinje cells within the cerebellum play an important role in the function of the cerebellum as they form the primary output of the cerebellar cortex to the nuclei, which then connects to the rest of the brain (35). Purkinje cells are known to be highly vulnerable to ischemic and excitotoxic insults as well as relying on myelinated axons to allow for efficient transmission of their signals (36, 37). The vulnerability of purkinje cells to hypoxic-ischemic-excitotoxic insults and reliance on myelinated axons suggests they may be highly vulnerable to hypoxic-ischemic events after preterm birth and consequently to impaired myelination.

It is well established that preterm birth, especially early preterm birth (26–32 weeks gestation), may result in cerebral white matter injury which causes profound neurodevelopmental consequences and pathology (38). White matter injury is understood to arise in the brains of preterm born neonates due to the susceptibility of immature, pre-oligodendrocytes to disturbed maturation caused by hypoxic-ischemic events which arise following preterm birth. The increased vulnerability of pre-oligodendrocytes to hypoxic-ischemic events arises due to a range of predisposing features (39). Pre-oligodendrocytes contain high levels of protein bound iron whilst also possessing relatively low levels of glutathione, a key antioxidant (40, 41). Hypoxia results in decreased oxidative phosphorylation which allows intracellular iron to become cytosolic, causing oxidative damage. Low levels of the antioxidant, glutathione amplify the impacts of oxidative damage in these pre-oligodendrocytes (42). Pre-oligodendrocytes also possess a high quantity of calcium permeable AMPA receptors, placing them at increased risk of undergoing excitotoxic calcium mediated apoptosis (43). Previous studies have established that preterm births in the period between 23 and 32 weeks gestation are most at risk of severe white matter injury due to the cerebrovascular immaturity present at these ages, contributing to increased hypoxia-ischemia and oxidative damage related to immature perfusion within the brain (44, 45). This disrupts oligodendrocyte maturation leading to less mature oligodendrocyte populations which in turn results in less myelin production (38). Despite the lower risk of extensive white matter injury, moderate-late gestation preterm born neonates still experience increased disruption of oligodendrocyte maturation and increased incidences of neurodevelopmental disorders. Neonates born moderate-late preterm may also experience hypoxic events in addition to losing exposure to placentally provided, protective neurosteroids (46, 47). The premature, abrupt loss of inhibitory neurosteroid supply may magnify episodes of hypoxia, leading to greater impairment of myelination and severe pathology. Moderate to late preterm born neonates have been found to exhibit myelination deficits with magnetic resonance imaging comparing 199 moderate-late preterm infants to 50 term born infants (38–44 weeks corrected postnatal age) finding that moderate-late preterm born infants had significant decreases in myelination as well as increased extracerebral space compared to term born infants (48). Despite not having as profound white matter injury compared to early preterm born counterparts, moderate-late preterm born infants still experience myelination deficits which likely contribute to their increased risk for neurodevelopmental disorders.

## 4 The importance of myelination

Myelination, as previously mentioned, continues into postnatal life, increasing its susceptibility to disruption following the adverse events of preterm birth (38, 49, 50). Oligodendrocytes begin as mitotically able, oligodendrocyte progenitor cells and progress through immature oligodendrocytes and pre-myelinating oligodendrocytes before maturing into myelinating oligodendrocytes (50). Each stage of maturation possesses specific properties pertaining to function and survivability (51).

Oligodendrocyte progenitor cells are mitotically able however, these cells have been shown to be highly sensitive to hypoxic, excitotoxic insult whilst immature oligodendrocytes are known to be more resilient to insult compared to later developmental oligodendrocyte stages (29, 30). The importance of correct and efficient maturation of oligodendrocytes is accentuated by the low numbers of mature, myelinating oligodendrocytes present during the late gestation, preterm birth period as disruptions to their development can significantly disrupt the development of myelinated axonal connections within the brain. Post-mortem analysis of 10 human preterm born neonates (GA 25–32 weeks) who had white matter lesions revealed that compared to term born controls, there were significant decreases in immunohistochemical staining for oligodendrocytes (O1), preoligodendrocytes (O4) and significant increases in apoptosis (caspase-3) (52).

Apoptosis of and delayed progression through the oligodendrocyte lineage due to hypoxic and excitotoxic damage caused by moderate to late preterm birth may potentiate myelin deficits. Hypoxia induced GABAergic signalling downregulation in mice, as evidenced by reduced expression of glutamic acid decarboxylase, a critical enzyme in GABA production, has been identified. Similar results have also been found in the post-mortem, preterm neonate study discussed above (52). This GABAergic signalling aberration has been shown to disrupt oligodendrocyte maturation in mice by causing an increased population of premature oligodendrocytes (OLIG2 fluorescence staining) whilst causing a decrease in the expression of mature oligodendrocytes (CC1 staining) when compared to normoxic controls. These aberrations were able to be remedied through the introduction of tiagabine, a drug which increases extracellular GABA concentrations by inhibiting GABA reuptake through blockade of GABA Transporter 1 (GAT1) (53).

The role of the cerebellum in the aetiology of ADHD in late preterm born groups is consistent with the late development of this region, particularly the process of myelination. Extensive studies have demonstrated cerebellar damage and dysfunction following very preterm (<32 completed weeks gestation) birth (54, 55). The development of the cerebellum does however continue to undergo critical periods of development through late gestation and continues developing into postnatal life (56). Myelination is one of the key processes which occurs into and beyond late gestation within the cerebellum. Preterm birth may arrest the development of the oligodendrocyte lineage, reducing the number of mature myelinating oligodendrocytes, thereby causing ongoing myelination impairment (57, 58). Myelination is critically important to the function of cerebellar Purkinjie cells. Purkinjie cells are GABAergic neurons which comprise the primary output pathway from the cerebellar cortex to the cerebellar nuclei and therefore play a key role in what information is conveyed by the cerebellum to other brain regions (59). Preterm birth associated damage leading to lower mature oligodendrocyte numbers and the associated reduced myelination could therefore have pronounced effects on cerebellar function and cellular development. Extensive connections from the cerebellum to the prefrontal cortex, amygdala and hippocampus, all areas which regulate emotional and executive functions, have been demonstrated (60–63). A potential role of the cerebellum in the preterm birth associated neurobehavioral conditions ADHD and anxiety, has also been shown through fMRI, BOLD and morphology studies in humans (60, 64–67). MRI studies have shown differences in the volume and morphologies of the cerebellum in ADHD. A study of 46 youths (ages 8–18) with ADHD found that these patients had significant decreases in surface volumes for several cerebellar lobules with further volume preserved warping analyses confirming reductions in volume in the underlying tissues in these regions. This study also found that the severity of symptoms correlated with more significant reductions in the volumes of lobules VIIIa and VIIIb (65). Meta-analyses have also reported that ADHD is consistently associated with volume abnormalities in cerebellar lobule IX, a lobule that has been established to play roles in attention and executive functions as well as functional connectivity (68–70).

## 5 Anxiety and attention disorders associated with impaired myelination and preterm birth

Anxiety disorders have been found to occur more commonly in moderate-late preterm born individuals compared to those born at term. Potjik et.al, examined a Dutch cohort of 995 moderate preterm children (32–35 weeks gestation) and 577 term born children, at 4 years of age, and found there were significantly increased odds ratios of 1.77 and 2.40 for total problems and internalising behaviours respectively. These investigators found there were marked sex differences with preterm born males having increased rates of elevated externalising behaviour problem scores whilst females were found to have increased elevated rates of internalising behaviour problem scores (71). Stene-Larsen et.al, found that late preterm birth resulted in a 47% increase in the odds of developing emotional problems in late preterm born girls (34–36 weeks gestation) at 36 months of age, when compared to term born girls (72). Intriguingly, this study of 43,297 children did not find any increase in the odds of developing emotional or behavioral problems in males however the authors did suggest this could be due to a difference in social development in males. This may have caused males to express these issues at older ages which were not assessed in the study, highlighting the importance of age and method of assessment.

The increased risk of developing ADHD following preterm birth has been shown to increase with declining gestational age in population based studies (73). Despite reduced risk with increased gestational age, this work showed that late preterm born individuals remain at significantly higher risk of developing ADHD than those born at term (73). The odds ratio for the use of ADHD medication was also significantly higher in the late preterm born cohort at 6–19 years of age when compared to children born between 39 and 41 (term) weeks of gestation (6). Interestingly, it was also found that “early term” births (37–38 weeks gestation) were also at increased risk of using ADHD medication (1.1 odds ratio) when compared to those born at term in this study (6). This is a very important finding given the increasing trend towards medical induction of labour at these times (72). This work is consistent with the effects of early term birth on ADHD-like, externalising behaviours in an Australian study. This study found that between the ages of 2–17 years of age, early term birth significantly increased the likelihood of externalising behaviours (1.4 odds ratio), compared to births occurring at 39 or more weeks gestation (74). The presence of an increased risk of requiring ADHD medication and developing externalising behaviours in individuals born at late preterm as well as early term further emphasises the potential for later neurobehavioral impairment in the moderate-late preterm period.

Impaired myelination has been observed in ADHD and anxiety disorders. Imaging studies comparing anxiety disorder sufferers and healthy controls have demonstrated differences in cerebellar volumes and activation between these groups (75). Myelination differences were also found in the cerebellum in a study of adults with panic disorder when compared to healthy control adults using a voxel-based morphometry analysis of cerebellar MRI images (76). Significantly reduced white matter volumes were found in the panic disorder group in the left inferior and cerebellar peduncle and right cerebellum, suggesting that anxiety disorders are impacted by impaired cerebellar myelination (77). It is well established that limbic structures such as the amygdala and hippocampus play a key role in anxiety behaviours (77). Growing evidence now supports the cerebellum playing a major role in integrating with several non-motor brain regions including the amygdala and hippocampus (78). This is further supported by a recent study of adult generalised anxiety disorder (GAD) patients with these patients showing altered cerebellar to amygdala connectivity (75). Myelination is vital for ensuring long range neuronal communication occurs effectively. The cerebellum forms reciprocal connections to the emotional processing centre of the brain, the limbic system (79, 80). Reduced cerebellar myelination could impair the communication between these regions, suggesting this is a potential mechanism by which impaired development of the cerebellum may influence the prevalence of these disorders.

## 6 Burden of childhood ADHD and anxiety

The devastating increase in incidences of ADHD and anxiety in children may be associated with greater numbers of late preterm births and early inductions of labour. ADHD has been reported in 10% of school aged boys with anxiety disorders being found to affect approximately 10% of all children (10, 11). The rates of these disorders are higher again in the preterm born population with the prevalence of ADHD found to increase with reduced gestation length. ADHD prevalence in preterm males and females combined are 12.1% for extremely preterm (22–27 weeks), 7.0% for moderately preterm (28–33 weeks), 5.7% for late preterm (34–36 weeks), 6.1% for all preterm (<37 weeks), 5.2% for early term (37–38 weeks), and 4.5% for full-term (39–41 weeks) (81). Anxiety prevalence in children born preterm have also been reported following meta-analyses to be 2 to 3-fold higher than children born at term (82, 83).

Estimates by the Australian ADHD Professionals Association (AADPA) predict that ADHD carries an AUD$20 billion social and economic health burden in Australia, 81% of which is determined by productivity loss due to the condition (84). Anxiety in an Australian study across all ages has been found to possess a significant social and economic health burden of AUD$4.7 billion (85). ADHD and anxiety in school aged children have both been associated with reduced productivity and academic achievement (86, 87). The impact on academic achievement of ADHD and anxiety sufferers places individuals at a greater risk of unemployment with a major effect on later life socioeconomic status. Later life criminal justice involvement is also associated with ADHD with studies showing that childhood ADHD increases juvenile and adulthood criminal justice involvement, with reports also showing high rates of criminal recidivism for individuals who are juvenile perpetrators (88, 89). The significant social and economic health burden on society and individuals due to childhood ADHD and anxiety emphasises the value of reducing the development of and effectively treating these conditions.

## 7 Role of neurosteroids in preterm birth cerebellar damage

The placenta not only provides nutrients to the developing fetus but also provides trophic hormones which are essential for supporting neurodevelopment, hormones which are abruptly and prematurely lost due to preterm birth. Neurosteroids and their immediate precursors produced by the placenta have key roles which influence neurodevelopment in the late-gestation period. Neurosteroids are psychoactive hormones which are provided by the placenta through metabolism of abundant, placental, progesterone production. Current evidence suggests allopregnanolone is one of the most critical neurosteroids for supporting neurodevelopment with extensive studies showing that this key neurosteroid acts on extra-synaptic GABA_A_ receptors (90). The resultant inhibitory neurosteroid action is critically important in neurodevelopment with available evidence suggesting that during the second half of human pregnancy, allopregnanolone confers an inhibitory, GABAergic “sleep like state” to the fetus by positively modulating GABA_A_ receptors from at least 24 weeks of human pregnancy. GABAergic stimulation is inhibitory from ∼60% of gestation in the long gestation species investigated (human, sheep, guinea pig). Allopregnanolone also provides a pro-myelinating effect which involves trophic action as well as neuroprotective effects by reducing neuronal excitation, preventing neonatal seizures and protecting against excitotoxic insult caused by neonatal hypoxia (91).

The impact of a premature decline in allopregnanolone concentrations has been examined experimentally in guinea pig and sheep pregnancies through the administration of finasteride (92). Finasteride prevents the synthesis of allopregnanolone from progesterone by inhibiting 5alpha-reductases (selective for type II and III), the key rate limiting enzyme in the synthesis pathway. In these studies, finasteride administration during the final third of guinea pig pregnancy (55–65 days gestation—term is ∼70 days) resulted in a 75% decrease in fetal brain allopregnanolone levels compared to controls. The fetuses exposed to the finasteride treatment were found to have significantly lower levels of myelination, as evidenced by lower myelin basic protein (MBP) staining in the CA1 region of the hippocampus (93). The impact of finasteride on both allopregnanolone levels and myelination within the fetal brain suggests that adequate allopregnanolone levels are required to provide a trophic impact on myelination. Adequate allopregnanolone levels thereby are critical for correct development of brain regions including the hippocampus with deficits in this region which governs memory and fear processing ultimately having major impacts on behaviour (92). Cumberland et.al further demonstrated the importance of allopregnanolone in the moderate-late preterm birth period in guinea pigs (94). This study found that finasteride administered from 60 days gestation (moderate preterm period) until term, again markedly decreased fetal allopregnanolone levels. At childhood equivalence age (PND20) it was found that female guinea pig offspring who were exposed to finasteride *in utero* displayed significantly increased incidences of neophobic-like responses to changes in environment in open field behavioural testing. These increased fear responses suggest that the level of exposure to allopregnanolone during the moderate-late preterm period play a critical role in regulating emotional problems in childhood. Acute finasteride administration to pregnant sheep during late gestation (129 days/87% gestation) produced a significant increase in fetal electrocorticogram and electromyogram recordings, including abnormal patterns of fetal activity. These findings in the sheep demonstrated that decreased allopregnanolone levels increase neuronal excitation and the results of the study further showed that this increase in neuronal activity places the fetus at higher risk for excitatory damage due to hypoxia (95).

The role of allopregnanolone in cerebellar development has been demonstrated in mice through the genetic knockdown of the gene encoding the allopregnanolone synthesis enzyme, 3*α*-hydroxysteroid dehydrogenase (96). In this study, the placental expression of 3*α*-hydroxysteroid dehydrogenase was eliminated, resulting in severely, significantly decreased allopregnanolone levels *in utero.* The mice exposed to the reduced placental supply of allopregnanolone were found to have significant, sex dependent myelination abnormalities in their cerebellums, as determined by western blot and immunofluorescent staining for MBP and MOG (myelin oligodendrocyte protein). The investigators also observed “autism spectrum disorder-like behaviours” in the male mice within this study. Interestingly, the administration of allopregnanolone and the selective GABA_A_ receptor agonist, muscimol were found to abolish these changes (96). Neurosteroids have been clearly implicated in white matter abnormalities and impaired myelination, factors which are known to impact cerebellar function and development. Additionally, it has been shown in mice that removing placental allopregnanolone results in cerebellar white matter and behavioural abnormalities which can be ameliorated through reintroduction of allopregnanolone or stimulation of the GABAergic pathway which allopregnanolone acts.

Current pharmacological treatments for ADHD and anxiety do not address the aetiology of these conditions and instead are symptomatic treatments. Tiagabine, muscimol and allopregnanolone have demonstrated the ability to restore oligodendrocyte maturation following hypoxia, potentially indicating the ability for GABAergic compounds to address the development of these disorders following preterm birth insult (53, 96). This is incredibly important clinically as the current “first-line” treatments for ADHD which include stimulant class drugs such as methylphenidate and dexamphetamine have a suite of undesirable effects and carry high risk for adverse outcomes. Adverse effects such as nausea, insomnia, nervousness and reduced appetite occur very commonly following the use of these stimulant medications (97, 98). Antidepressants and alpha agonists are also used in the treatment of ADHD and these come with their own adverse effect profiles and also aren't able to modify the course of this pathology (99, 100). Antidepressant drugs are the current first line pharmacotherapy for the treatment of anxiety and this class of drug commonly causes side effects such as nausea, insomnia, decreased sex drive and agitation (101). Current pharmacotherapies for both ADHD and anxiety are also impacted by adherence issues which arise due to the chronicity of treatment as well as the persistent adverse effects conferred by these drugs. ADHD medication adherence has been reported being as low as 36% after 5 years of treatment and antidepressant drug therapies have been found to hold only 43% adherence in anxiety sufferers after 6 months (102–104). Prophylactic therapies which replace allopregnanolone or modulate the GABAergic system, if given in the immediate, postnatal period following preterm birth would not be subject to chronic-use compliance issues and could effectively address the impaired development of the cerebellum which contributes to the development of these disorders.

## 8 Conclusion

Preterm birth is commonly associated with hypoxic events, insults which have a high propensity to cause excitatory damage to the dense neuronal populations of the cerebellum. The cerebellum also relies heavily on myelinated, GABAergic, purkinje cells to communicate with other regions of the brain. Hypoxic and excitotoxic events are known to preferentially inhibit the maturation of oligodendrocytes, thereby reducing myelination in the cerebellum following preterm birth. It is now established that the cerebellum plays a role in non-motor processes such as attention, emotional processing, cognition and has been implicated in neurobehavioral disorders such as ADHD and anxiety.

The impact of preterm birth on the cerebellum and the relationship between preterm birth and neurobehavioral conditions suggests that the effects on the development of the cerebellum have important roles in these conditions. Consideration of the vulnerability of the cerebellum to moderate-late preterm birth associated insults represents a potential pathway for therapies to be identified which may help to reduce the risk of developing neurobehavioral conditions following preterm birth. The current treatments for these disorders are plagued by adverse effects and adherence issues making a potential prophylactic treatment a highly valuable clinical tool which could alleviate the burden of these disorders. Current evidence suggests treatments which target the GABAergic system or reduce excitatory transmission in the neonatal preterm period may provide effective protection against the neurodevelopmental deficits contributing to these disorders.
